# Scellpam: an R package/C++ library to perform parallel partitioning around medoids on scRNAseq data sets

**DOI:** 10.1186/s12859-023-05471-1

**Published:** 2023-09-14

**Authors:** Juan Domingo, Teresa Leon, Esther Dura

**Affiliations:** 1 Department of Informatics, ETSE, University of Valencia, Avda. de la Universidad, s/n, 46100 Burjassot, Valencia Spain; 2 Department of Statistics and Operations Research, University of Valencia, Avda. Vicente Andres Estelles, 46100 Burjassot, Valencia Spain

**Keywords:** Partitioning around medoids, Parallel implementation, Dissimilarity matrix, Large datasets, Single-cell RNA-seq analysis

## Abstract

**Background:**

Partitioning around medoids (PAM) is one of the most widely used and successful clustering method in many fields. One of its key advantages is that it only requires a distance or a dissimilarity between the individuals, and the fact that cluster centers are actual points in the data set means they can be taken as reliable representatives of their classes. However, its wider application is hampered by the large amount of memory needed to store the distance matrix (quadratic on the number of individuals) and also by the high computational cost of computing such distance matrix and, less importantly, by the cost of the clustering algorithm itself.

**Results:**

Therefore, new software has been provided that addresses these issues. This software, provided under GPL license and usable as either an R package or a C++ library, calculates in parallel the distance matrix for different distances/dissimilarities ($L_1$, $L_2$, Pearson, cosine and weighted Euclidean) and also implements a parallel fast version of PAM (FASTPAM1) using any data type to reduce memory usage. Moreover, the parallel implementation uses all the cores available in modern computers which greatly reduces the execution time. Besides its general application, the software is especially useful for processing data of single cell experiments. It has been tested in problems including clustering of single cell experiments with up to 289,000 cells with the expression of about 29,000 genes per cell.

**Conclusions:**

Comparisons with other current packages in terms of execution time have been made. The method greatly outperforms the available R packages for distance matrix calculation and also improves the packages that implement the PAM itself. The software is available as an R package at https://CRAN.R-project.org/package=scellpam and as C++ libraries at https://github.com/JdMDE/jmatlib and https://github.com/JdMDE/ppamlib The package is useful for single cell RNA-seq studies but it is also applicable in other contexts where clustering of large data sets is required.

**Supplementary Information:**

The online version contains supplementary material available at 10.1186/s12859-023-05471-1.

## Background

Clustering plays a significant role in handling large datasets, particularly in the pipelines of information processing within Bioinformatics [1].

Many fields, including bioinformatics, face challenges that involve analyzing large/huge datasets consisting of numerous individuals *n* (points or objects) and features *d* (or dimensions). The number of final clusters, *k*, being a critical aspect that needs to be considered.

Clustering large data sets requires either the choice of an algorithm with a low memory footprint and a low computational cost, or, alternatively, the availability of clever (and if possible, parallel) implementations of the existing methods. One of the subareas that could benefit most from these improvements is the analysis of single cell sequencing experiments. Modern sequencing devices are able to process a number of cells approaching the range of 100,000 and aggregation of data from several experiments produces even much larger datasets. See [2] for a review and a performance evaluation of clustering methods for single-cell RNA-seq data.

Partitioning around medoids (PAM) [3] is a widely used, classic clustering method that can produce highly reliable solutions. However, both its memory and its computational cost for clustering a large number of individuals is too high for current computers unless an efficient implementation of the method is used [4]. PAM is based exclusively on distances^1^ between points. Once the desired number of groups to find *k* is decided, it seeks to find an optimal set *M* of *k* representatives, $M=\{m_1,\ldots ,m_k\}$ selected from the initial set so that the total deviation (TD), defined as follows, is minimized.

$$\begin{aligned} TD=\sum _{i=1}^k \sum _{x_c \in C_i}dist(x_c,m_i) \end{aligned}$$

TD is the sum of distances of each point $x_c\in C_i$ to the medoid $m_i$ of its cluster. The medoid of a set is the object with the smallest sum of distances to all other objects in the set:

$${\text {medoid}} (C):=arg min_{x_m\in C} \sum _{x_c \in C} d(x_c,x_m)$$

PAM is made up of two algorithms: the first one is used to select the initial clustering, and the second one is a swapping algorithm that iteratively improves the clustering towards a local optimum.

PAM, also known as K-medoids clustering method, has not been widely applied due to two main issues: its quadratic memory cost ($\mathcal {O}(n(n+1)/2$) and its high computational cost. The first challenge arises from the need to calculate the distance matrix, which typically takes $\mathcal {O}(n^2d)$ time. Then, initial medoids must be identified; doing this using the BUILD method can take $\mathcal {O}(n^2k)$ time. Finally, the optimization phase involves swapping between a point of the current set of medoids and the rest of the points, with a cost of $\mathcal {O}{(k(n-k)^2)}$ per iteration, even when using the FASTPAM1 variant proposed in [4].

The implemented software we propose tackles these problems in two ways. In the first place it allows the data, including distance matrices, to be stored with any data type and with matrices of almost arbitrary size (matrix indices are 32-bit unsigned integers so limits are only based on the physical amount of memory). This might seem obvious; however, is not easy for example in R implementations, where the matrices are by default double type and some packages use indices which are 16-bit integers. Also, storing of matrices with a high density of zeros is implemented as sparse, and only the lower diagonal part of symmetric matrices is stored. Furthermore, the calculation of the distance matrix, the initialization step using BUILD algorithm and the swapping step of FASTPAM1 have been implemented in parallel. As will be shown in Results section, this significantly reduces the computation time in all cases, especially as the number of available processors/cores increases. From now on, we will assume that all the needed data have been loaded and are accessible in the memory of the computer. While it is technically feasible to implement versions of the algorithms that load data from storage media on demand (e.g., from disk), this approach typically results in extremely slow execution times and is not considered practical unless no other options are available.

## Implementation

A C++ library (jmatlib) allows the storage on disk, reading and writing of 2D matrices as binary data including a header and optional metadata (names of rows and/or columns and a comment). It is possible to access the characteristics of a given matrix simply by reading the header, without the need to load the complete matrix. Also, a matrix can be full, sparse or symmetric and can hold any data type, from char to long double.

A second C++ library (ppamlib) contains the parallel implementation of the distance matrix calculation (currently, five different distances/dissimilarities are available: Euclidean (*L*2), Manhattan/City block (*L*1), Pearson dissimilarity coefficient, cosine dissimilarity and weighted Euclidean distance). Furthermore, the implementation of the algorithm takes into account whether the input data is sparse, allowing it to ignore the zeros during the distance calculation process.

This library also contains the implementation of the PAM algorithm. Two algorithms are available for the initialization phase: BUILD (deterministic) and LAB (random). The optimization (swapping) phase uses the algorithm called FASTPAM1 (see [4]).

This library also includes the calculation in parallel of the mean silhouette width [5] of a cluster which is a good measure of cluster quality and also a guidance to select the number of groups.

Finally, the R package scellpam which provides the interaction with these libraries from R is provided to allow bioscientists to load the single cell data (count matrices) in the formats habitually used in the field, such as the SingleCellExperiment (sce) class of Bioconductor [6], the format used by Seurat [7–10], or a full or sparse R matrix directly, so that distance calculation and PAM can be easily applied. It is important to note that, while the matrix and distance/PAM libraries can also be accessed from the R packages jmatrix and parallelpam, the scellpam package includes the functionality of these two, so it is the only one that needs to be installed for biological analysis; the others are provided mainly for researchers in other fields.

The normal workflow in the use of this software for the analysis of single cell experiments involves the reading of the count matrix with the package that understands its format and writing it into JMatrix format. For instance, a simple count matrix stored in a .rds file written by the Seurat package is read as:
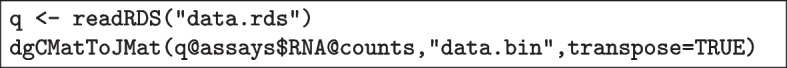


Alternatively, reading from a package which works with the SingleCellExperiment (.sce) class such as DuoClustering [11] is done as,
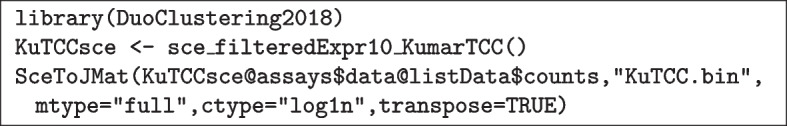


and then distance matrix is obtained from the counts, and PAM is applied with
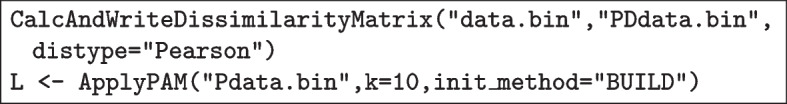


The package also includes a function called BuildAbundanceMatrix(), which builds and returns the abundance matrix. This function can also generate labels for the initial data if they are associated with experimental conditions.

## Results

The first test of the package has been done using gene expression data from a single-cell sequencing experiment on human endometrium across the natural menstrual cycle with $N=71,032$ cells due to Wang [12]. Its NCBI GEO accession number is GSE111976.

It has to be mentioned that the first two authors of this paper are also the co-authors of an article submitted to this journal in which this and other datasets mentioned later are analyzed using scellpam; this work is available for review in [13]. In contrast to the software paper being presented here, the aforementioned article focuses on the study of the dynamics of endometrial remodeling through the menstrual cycle using clustering analysis and a cell abundance analysis of the data set [12]. In [13] we were mainly interested in comparing the performance of *s*cellpam with other packages used in the context of scRNA-seq, but in this one we compare our implementation with R packages that compute distances and dissimilarities, as well as with R packages which implement K-medoids.

All the experiments carried out in this work aim to highlight the strengths of the implementation. Firstly, we discuss the computational issues related to the calculation of the distance matrices for all the aforementioned data sets, and then a second experiment to apply the PAM clustering method using the $L_2$ distance metric is discussed. Also, the comparison with other packages is done taking as a basis the Wang data set or subsets of it. In some experiments, all 71,032 cells were used, while in others, random samples were selected from the total set of cells. Specifically, three random samples were selected from the entire set of cells, corresponding to one eighth, one quarter, and one half of the total number of cells (8879, 17,758, and 35,516 cells, respectively).

This experimental setup aimed to measure the performance of the implementation using different numbers of threads. The tests were carried out on a machine with 64 cores (an AMD Ryzen Threadripper 3990X) and 128 GiB of RAM. A first test was run without using any threads, followed by tests using 8, 32, 64, and 128 threads. The trial with 128 threads utilized the hyperthreading capability of the cores. The purpose of these tests was to determine how the implementation scaled with increasing numbers of threads, and how effectively it utilized the available processing power. In all cases the machine was exclusively devoted to this task, and nothing else, apart from the kernel and necessary daemons were running. Memory swapping to disk was explicitly forbidden.

### Distance calculation

The distances between the cells were calculated using the five provided possibilities ($L_1$ and $L_2$ distances, as well as the Pearson correlation coefficient dissimilarity, cosine dissimilarity and weighted Euclidean distance). The computation time for distance calculation using the *s*cellpam package was compared with other available packages, including *stats* which is part of R core ([14]), *cluster* ([15]), *parallelDist* ([16]), and *amap* ([17]). The *stats* package is a basic R package that includes the dist function, while *cluster* is a popular package for basic clustering algorithms that provides the daisy function. *parallelDist* is a package that offers a fast parallelized alternative to R’s native dist and provides the pdist function. *amap* is a package for standard hierarchical clustering and k-means, which includes the Dist function for parallelization.

In cluster analysis packages, it is common to represent individuals as rows and features as columns in the input matrix, which is also the approach adopted in this study. However, this differs from the typical convention in single-cell analysis packages, where the representation is reversed. Despite this difference, this is not a problem because the functions of *scellpam* allow for the option to transpose the input matrix if desired, thus accommodating both conventions. Moreover, most packages require the data matrix to be loaded in R memory as a matrix of doubles, but the input format of the data matrix for *s*cellpam is binary and it can be either float or double, and a full or sparse matrix. The package has implemented special procedures to calculate the distance matrix when data are sparse, which is particularly important for single cell RNA-seq data, as it can further reduce the time. While R offers the use of sparse matrices in its matrix package, most packages for calculating distances only operate with full matrices. Therefore, *s*cellpam has been compared to other packages using both a sparse matrix of floats and a full matrix of doubles to make the comparisons fair.

The result for L2 distance calculation with serial execution (no threads used in the programs) are shown in Table 1. In these and successive tables the best result for each experiment is highlighted using bold font.

**Table 1:** Time in seconds for distance matrix calculation, L2 distance, serial execution

	R package					
Sample	amap	parallelDist	cluster	stats	scellpam	scellpam
size	(Dist)	(pdist)	(dist)	(daisy)	(double, full)	(float, sparse)
8879	20980	4469	10338	10477	4049	**2079**
17758	155651	68326	59804	59467	16073	**8200**
35516	196906	273738	541886	516625	64382	**32956**
71032	($*1$)	551527	($*2$)	2214564	256678	**131474**

($*1$) Error in Dist(data, method = ”euclidean”, nbproc = 128, diag = FALSE: Long vectors (argument 4) are not supported in.C

($*2$) Error in daisy(data, metric = dtype, stand = FALSE): long vectors (argument 11) are not supported in.Fortran Execution halted

As previously mentioned, a fair comparison for the general case should involve full matrices in double precision. Our package outperforms the others in terms of speed, except for the *parallelDist* package, which is comparable for a small number of individuals (taking 4469 s compared to our 4049 s for 8879 cells). However, its execution time significantly increases for larger numbers of cells (taking 68326 s compared to our 16073 s for 17,758 cells). It is worth noting that in our case, the time required increases quadratically with the number of individuals *n*, since the number of elements in the distance matrix is $n(n+1)/2$. This is the expected behavior, but it does not hold for the other packages. Furthermore, two of them (amap and cluster) are incapable of handling more than 65,536 individuals and produce the errors displayed below the table.

In the case of this sparse matrix, our algorithm avoids calculating the squared difference of components when both components are 0, which proves to be advantageous, as evidenced by the comparison of the last two columns.

The results for parallel execution in the packages that allow this (ours, *amap* and *parallelDist*) are summarized in Table 2.

**Table 2:** Time in seconds for distance matrix calculation, L2 distance, multithread execution

R package	Sample	Number of threads			
	size	8	32	64	128
scellpam (sparse, float)	8879	**283.1**	**76.0**	**44.4**	**32.5**
scellpam (full, double)		551.8	154.7	115.5	107.4
amap		2775.7	1570.4	1815.7	4978.2
parallelDist		1963.0	826.7	1665.9	4148.3
scellpam (sparse, float)	17,758	**1122.1**	**300.9**	**174.8**	**129.1**
scellpam (full, double)		2170.3	601.3	431.6	408.6
amap		14673.0	18077.3	25365.7	31961.7
parallelDist		8794.5	10021.2	22694.2	31734.1
scellpam (sparse, float)	35,516	**4494.1**	**1192.6**	**692.4**	**508.6**
scellpam (full, double)		8774.1	2408.1	1694.4	1625.6
amap		69393.0	92699.9	87231.0	63080.0
parallelDist		36236.8	5751.6	104415.0	119098.0
scellpam (sparse, float)	71,032	**17933.0**	**4730.3**	**2736.5**	**1994.7**
scellpam (full, double)		35240.1	9534.3	6694.4	6534.9
amap		$(*1)$	$(*1)$	$(*1)$	$(*1)$
parallelDist		159231.0	545900.0	413000.0	489916.0

($*1$) Error in Dist(data, method = ”euclidean”, nbproc = 128, diag = FALSE: Long vectors (argument 4) are not supported in.C

The results obtained from the *parallelDist* package for 32, 64, and 128 threads are not exact, but rather estimated. These estimates were based on the number of distances that had been effectively calculated at regular intervals until execution was interrupted after four days. To achieve this, small modifications to the source code were necessary to display progress from time to time.

In the experiment involving parallel execution, our package performs significantly better, particularly when using float and sparse matrices. The differences between our package and the others are even more pronounced than in serial execution. Furthermore, the behavior of our package is consistent: execution time increases with the number of cells and decreases with the number of threads/cores, although not exactly linearly, possibly due to issues with the maintenance of cache memory coherence. This effect is particularly noticeable with 128 threads compared to 64, as using 128 threads on our computer involves the use of hyperthreading, which is not ideal for numerically intensive algorithms that rarely use input/output operations.

In the case of the other packages, all of them are consistent with respect to the number of cells (time increases with number, as expected), but not with respect to the number of threads. Both the *amap* and *parallelDist* packages achieve their best results with 32 threads, which is surprising since the programs run on a machine with 64 physical cores, which means that no core should hold more than one thread.

### Application of PAM

The second experiment involved applying the PAM clustering method using the L2 distance metric.

Similar to the previous experiment, results were obtained using the same data and applying the PAM algorithm implemented in several R packages, namely:

- *c*luster [15] used in the former experiment which provides the function pam.
- *f*astkmedoids [18] which provides a function called pam, too.
- *C*lusterR [19, 20] which provides the function Cluster_Medoids
- *s*cellpam

We also experimented with the *kmed* package [21], which uses a different algorithm that is theoretically faster, but it produced suboptimal results compared to the other packages (i.e., with a larger value of the optimization function).

The input to PAM consists of two arguments: a distance/dissimilarity matrix (i.e., an $n\times n$ symmetric matrix) and the number of medoids to be found. In our package, the input is provided as a matrix stored in our binary format (symmetric jmatrix) on disk. In the other packages, the input is either a standard R matrix or a vector with the elements in the lower diagonal of the symmetric matrix ordered by columns. We chose 30 medoids for the test, as this number of clusters is reasonable for this dataset based on biological knowledge.

The function pam in the *cluster* package implements several variants of the PAM algorithm which can be chosen with the parameter pamonce=.... With respect to our package, the variant we implemented is the fastest of those used by the cluster package and therefore is also the one used for comparison (pamonce=5). Indeed, the Help section of pam:cluster explicitly states “’pamonce=5’ adds minor optimizations copied from the ‘pamonce=2’ approach, and is expected to be the fastest of the ‘FastPam’ variants included”.

It is worth noting that to ensure a fair comparison all the packages were allowed to run through both phases of the algorithm, namely the BUILD phase and the optimization phase. The BUILD phase involves the selection of initial medoids using the BUILD method described in [4]. The optimization phase involves swapping medoids until the optimization function can no longer be improved. Although the number of iterations required to reach this state in some cases differ among packages, the final results (i.e., the set of medoids found) are either the same or equivalent. Equivalent means that in some cases the software finds a set of medoids that differ by two or three from the standard result, but with an optimization function value with negligible differences between both implementations. This is another local minimum that is equivalent to the most commonly found one. See detailed comments in “Test with bigger data sets section”. The fact that different implementations yield the same or equivalent results provides strong evidence for the correctness of our implementation, especially the use of floats for the distances.

Spent time in seconds, with the same computer and same conditions as in the former experiment, is shown in Table 3 for serial execution (one thread).

**Table 3:** Time in seconds for complete serial execution of PAM (initialization with BUILD + optimization)

Sample	Package			
Size	Cluster	Fastkmedoids	ClusterR	Scellpam
8879	14.2	13.0	290.1	**12.16**
17,758	60.7	57.0	977.0	**52.0**
35,516	258.0	**249.2**	4266.8	271.8
71,032	NA	SF	19468.7	1**764.0**

NA: package indicates with a message that no more than 65,535 individuals are allowed

SF: Segmentation fault

Note: Cluster package used with parameter pamonce=5

Regarding parallel execution, only the *ClusterR* package along with our package currently implement a parallel version of PAM, allowing us to compare their performance using different numbers of threads: 8, 32, 64, and 128. As before, the use of 128 threads involves hyperthreading on our machine’s 64 cores. The results are presented in Table 4, where the columns labeled ClR correspond to the *ClusterR* package and the columns labeled scp correspond to our package, *s*cellpam.

**Table 4:** Time in seconds for complete parallel execution of PAM (initialization with BUILD + optimization)

	Number of threads							
Sample	8		32		64		128	
Size	ClR	scp	ClR	scp	ClR	scp	ClR	scp
8879	35.5	**2.8**	13.1	**1.1**	9.9	**0.7**	16.97	**0.6**
17,758	142.7	**9.6**	54.9	**3.2**	94.8	**2.2**	125.1	**2.1**
35,516	620.1	**49.8**	447.7	**16.8**	503.8	**13.9**	538.5	**17.0**
71,032	3107.1	**560.6**	2133.5	**210.6**	2183.0	**197.1**	2300.4	**255.2**

The columns labeled *ClR* correspond to the ClusterR package, while the columns labeled *scp* correspond to the *s*cellpam package introduced in this paper

Times for serial execution are quite similar for our package and for *cluster* and *fastkmedoids*. Maximum differences show improvements of 17% and 16% for 8879 and 17,758 individuals, respectively, of our package with respect to *cluster*, but the *fastkmedoids* package improves on our time by 9% in 35,516 individuals, as does the *cluster* package by 5%. The problem arises with the whole sample of 71,032 individuals as these packages cannot cope with this. On the other hand, package *ClusterR* shows much higher serial execution times than ours, in the best case (71,032 individuals) it is 11.3 times slower.

In our experiment, parallel execution showed a significant improvement, but the time reduction was not proportional to the number of threads. The lack of proportionality was partly due to issues with maintaining cache memory coherence, and partly due to how the original algorithm was parallelized. Further improvements are needed to address this issue.

The *ClusterR* package had longer execution times than our implementation, but it still performed better than serial execution. In its best case (71,032 individuals with 8 threads), we were 5.5 times faster than *ClusterR*, and in its worst case (8879 individuals with 128 threads), we were 30 times faster.

Both our implementation and *ClusterR* were consistent when increasing the number of individuals, with higher numbers resulting in longer computation times. Our implementation was also consistent in behavior when changing the number of threads, except when using hyperthreading. We do not recommend using hyperthreading with our implementation of PAM. *ClusterR* exhibited similar behavior when using hyperthreading, but it also behaved strangely, for example increasing computation times between 32 and 64 threads for all sample sizes except 8879 individuals.

Finally, and with respect to memory, our package uses half of the memory used by the others, since it can operate with float numbers (4 bytes each) instead of double-precision numbers. For the used set (71,032 cells) this means 9.39 GiB instead of 18.79 GiB. In a machine with 128 GiB of RAM, this allows the possibility of accommodating a distance matrix of about 250,000 individuals instead of 180,000 if double were used. Nevertheless, note that tests should be done with a sub sample to check that the loss of precision does not change the final results (i.e.: the final set of medoids given by PAM). See more detailed tests in “Test with bigger data sets” section.

### Test with bigger data sets

Scalability of the package has been tested applying its functions to other data sets of single cell RNA sequencing with a larger number of cells, and to a data set with a different type of data. Namely:

- The data set used in former experiments, scRNA-seq on human endometrium throughout the natural menstrual cycle; $N=71,032 cells$. NCBI GEO accession number GSE111976 [12]. This is denoted in the tables as Wang. Even though it has been used in the former experiments, it is also included here since other distances/dissimilarities have been calculated for it.
- FMA, a Data set For Music Analysis, made available in the UCI Machine Learning Repository (https://archive.ics.uci.edu/) as detailed in [22] whose meaning and content is explained in [23]. It has $N=106,574$ instances with 518 numerical attributes each and it is included as an example of non-biological data set. Denoted in tables as FMA.
- scRNA-seq of endometrial superficial biopsies; $N=100,307$ cells denoted in the tables as Garcia. ArrayExpress accession number E-MTAB-10287 [24].
- scRNA-seq of endometriosis; $N=118,144 cells$. NCBI GEO accession number GSE213216 [25] denoted in the tables as Fonseca.
- A combination of the previous three scRNA data sets; $N=289,483$ cells denoted as Merge.

The following tests were carried out with the same computer and in identical conditions as those in the previous sections. First, the calculation of the different distances/dissimilarities was done for all the data sets using 4,000 features for the single cell data (the most highly variable genes normalized with method *log*1*n*, i.e.: to the logarithm of number of counts plus one) or all the provided features (518) for the FMA data set. Moreover, the fastest possibilities offered by the package were used: data represented as float values, use of sparse matrices for the scRNA data sets (the FMA is made of full data) and the maximum number of threads (in our machine, 128). The results are shown in table 5 in terms of spent time in s. The next two tables partly coincide with Tables 4 and 5 in [13], even though that paper has a mainly biological approach it also presents some computational results using the data sets [24, 25] and Merge.

**Table 5:** Time in s for calculation of several distances/dissimilarities in large data sets

	Number of					
Data set	Instances	$L_1$	$L_2$	Pearson	Cosine	Weighted Euc.
Wang	71,032	324.40	318.53	228.22	285.77	316.04
Garcia	100,307	772.94	761.19	459.19	665.20	753.25
FMA	106,574	165.92	168.05	155.05	162.09	166.67
Fonseca	118,144	663.12	654.61	585.62	534.70	654.17
Merge	289,483	5434.67	5363.10	3777.32	4647.69	5311.57

The increase in computation time is not so clearly quadratic with the number of individuals this time, indicating a dependency on the specific characteristics of the data being processed. The FMA data set is clearly different as it is of a much smaller dimension (518 features per datum vs. the 4000 features/gene expressions chosen for the single cell data sets) and it is not sparse. In single cell data sets all metrics/dissimilarities are calculated faster for the Fonseca than for the Garcia data set. This apparent paradox is related with the number of zeros (i.e.: the degree of sparsity) of the data, which is greater for the Fonseca data set. In order to verify this, we conducted tests on both data sets using full matrices and evaluated the $L_1$ and $L_2$ metrics. Our implementation with full matrices computes the difference between coordinates even if they were both zero. The results consistently showed that the $L_1$ metric took 1360.55 seconds for the Garcia data set and 1495.14 seconds for the Fonseca data set. The $L_2$ metric exhibited a similar behavior, with computation times of 1367.77 seconds for Garcia and 1519.06 seconds for Fonseca.

A second test involved applying the PAM algorithm to these datasets, including both phases: initialization with BUILD and medoid swap for optimization with FASTPAM1. The distance matrices used were the ones generated in the previous experiment. Table 6 displays the corresponding time spent for each case always using 30 medoids.

**Table 6:** Total time in s and number of iterations until convergence of the medoid swap phase for PAM execution with different distances/dissimilarities in large data sets

Data set	Number of instances	$L_1$		$L_2$		Pearson		cosine		Weighted Euc.	
Wang	71,032	229.19	5	255.74	9	314.05	18	287.35	13	222.97	4
Garcia	100,307	536.79	8	565.61	10	607.45	13	553.23	9	441.13	1
FMA	106,574	816.23	21	1140.72	40	1061.32	36	924.59	29	682.64	13
Fonseca	118,144	727.01	6	747.95	7	809.87	10	928.54	15	662.96	0
Merge	289,483	8393.42	13	9158.63	17	9364.45	18	8618.01	14	6286.18	2

The time used to execute PAM depends on the number of individuals, increasing with this, but there is no clear pattern that relates this with the used metrics. This is because the duration of the second phase of the method (swapping medoids with FASTPAM1) depends on the number of iterations, which in turn depends on the actual data and on how well the used metric captures the inherent clustering structure, if any, with the chosen number of medoids. Indeed, FMA sound data are much less structured, and the number of iterations until convergence in the second phase of PAM is greater than for the single cell data of comparable sizes, as Table 6 shows.

As stated before, the single cell data biological interpretation of the resulting clusters, along with the calculation of silhouette, must guide the selection of the metric and other possible choices for the preparation of the inputs used in the biological pipelines of the data (such as normalization method, etc.).

Nevertheless, it must be noted that even with the largest set of data (Merge) the complete process of dissimilarity matrix calculation plus PAM in the slowest case (Pearson, 5363 s plus 9158 s) gives a total of 4 h and two minutes, a long but still manageable time.

Indeed, the main limitation of the method, given the current sizes of the single cell sequencing experiments is the amount of memory. A data set of 1,000,000 instances which uses float for the distance matrix storage would need about 1.8 TiB. This is not yet common on machines currently used by most research groups but it is within the reach of current technology.

Finally, a test was carried out to verify the feasibility of using float instead of double precision numbers to store the distance matrix and to simultaneously verify the accuracy of the software. 100 samples of 10,000 individuals each were randomly taken from the Wang data set. For each sample, the PAM with Pearson dissimilarity was applied by *s*cellpam first using float for the dissimilarity matrix and then using double, and the results were compared with the standard implementation in the *c*luster package. The tests with float yielded no difference in the optimization function (sum of distances to medoids) for 63 of the 100 tests. For the others, its maximum relative error was $0.52\%$. Similarly, the tests with double precision values yielded identical results in 91 out of the 100 tests, the maximum relative error for the 9 cases left being $0.22\%$. In this second case, the reason for the differences are ties in the distances (up to the level of precision of the representation) that can motivate the algorithm to choose one swap instead of an equivalent one, depending on the implementation. The complete results can be seen in the Additional file 1.

In each case the user should decide if this error committed using float is acceptable for the intended use of the clusters, balancing it with respect to the reduction in memory usage.

## Conclusion

A new implementation of distance matrix calculation and the PAM algorithm has been developed, which can be used from R, or as a command line tool, and is publicly available under the GPL. This implementation offers several advantages over previous ones. For example, it allows arbitrary data types and uses only the memory strictly needed, it is not limited by the number of points or features, and it runs in parallel in a way that is transparent to the user, resulting in significantly reduced execution times that make the method applicable for the current typical sizes of single cell experiments.

The biological implications of the use of PAM were highlighted in [13], where the method and this package were used to distinguish subpopulations of epithelium, stroma, endothelium, perivascular cells and immune cells of the endometrium during the period of time known as the window of implantation of the embryo, but it is worth noting that while the original purpose of the package is to cluster single-cell RNA-seq data sets, the R packages/C++ libraries presented in this work can also be used for other types of data.

### Availability and requirements

Project name: scellpam Project home page: https://cran.r-project.org/web/packages/scellpam/index.html Operating system(s): Platform independent. Programming language: R and C++. Other requirements: R ver. 4.1 or later with packages Rcpp (ver. 1.0.8 or later), memuse (ver. 4.2.1 or later) and cluster (ver. 2.1.4 or later). C++ compiler, as needed by Rcpp. License: GNU GPL. Restrictions to use by non-academics: No restrictions applicable.

### Supplementary Information

**Additional file 1:** Difference of results of PAM clustering using float and double as implementation type.

## Data Availability

The data sets analyzed during the current study are available in the GEO and ArrayExpress repositories: scRNA-seq on human endometrium across the natural menstrual cycle; $N=71,032 cells$. NCBI GEO accession number GSE111976 [12]. scRNA-seq of endometrial superficial biopsies; $N=100,307$ cells. ArrayExpress accession number E-MTAB-10287 [24]. scRNA-seq of endometriosis; $N=118,144 cells$. NCBI GEO accession number GSE213216 [25]. The data of the FMA are available in the UCI Machine Learning Repository (https://archive.ics.uci.edu/) In this study we did not use any kind of cell lines.

## Abbreviations

scRNA-seq: Single-cell RNA sequencing
PAM: Partitioning around medoids
PAM-BS: PAM with BUILD$+$SWAP
TD: Total deviation
ARI: Adjusted Rand index
GiB: Gibibyte ($2^{30}$ bytes)
GPL: GNU General Public License
NA: Not available
SF: Segmentation fault

## Footnotes

1 From now on we will use the term distance to refer to both, distances or dissimilarities which are not metrics except when strictly needed.
