# Supplementary material for: Scellpam: an R package/C++ library to perform parallel partitioning around medoids on scRNAseq data sets
Source: BMC Bioinformatics. 2023 Sep 14;24:342. doi: 10.1186/s12859-023-05471-1 (PMC10503022; doi:10.1186/s12859-023-05471-1)
Supplement: Supplementary file 1 — Additional file 1: Difference of results of PAM clustering using float and double as implementation type. [file 12859_2023_5471_MOESM1_ESM.pdf]

Supplementary material to paper "scellpam: an R package/C++ library to perform parallel Partitioning Around Medoids on scRNAseq data sets"

J. Domingo, T. Leon, E. Dura

Results of the tests for consistency/accuracy of the results.

100 random samples of 10,000 instances (cells) each were chosen from the data set Wang that comprises 71,032 cells.

For each sample, the L_2 distance matrix was calculated using either $f$loat or $d$ouble as data types. Later, the partitioning around medoids (PAM) clustering algorithm was applied using the $s$cellpam and the $c$luster package. This generates two clusterings (partitions of the set of instances). To check to what extent both partitions differ the Adjusted Rand Index (ARI) between them was calculated. If $P = \{1, 2, \ldots s\}$ and $P^* = \{1, \ldots, r\}$ denote two partitions of a given data set the ARI(P, P^*) is defined as

$$\frac{Index - Expected\_index}{Max\_index - Expected\_index} = \frac{\sum_i \sum_j \binom{n_{ij}}{2} - \left[\sum_i \binom{a_i}{2} \sum_j \binom{b_j}{2}\right] / \binom{n}{2}}{\frac{1}{2}\left[\sum_i \binom{a_i}{2} + \sum_j \binom{b_j}{2}\right] - \left[\sum_i \binom{a_i}{2} \sum_j \binom{b_j}{2}\right] / \binom{n}{2}}$$

where n_{ij} is the number of individuals belonging to class i in the first clustering (P) and to class j in the second one (P^*), a_i ($1 \leq i \leq r$) is the number of individuals in class i in P^* and b_j ($1 \leq j \leq s$)is the number of individuals in class j in P.

ARI value is equal to 1 exclusively for identical partitions.

The tables Supplementary Table 1 and Supplementary Table 2 show in each column, in this order:

- The number of sample (1 to 100)
- The ARI between the partitions obtained by $s$cellpam and $c$luster
- The value of the difference between the objective function (TD) of both clusterings, i.e.$TD_{cluster} - TD_{scellpam}$
- The relative error, in %, i.e. $\frac{100 \cdot |TD_{cluster} - TD_{scellpam}|}{(TD_{clus} + TD_{scellpam})/2}$

exclusively for those samples in which ARI is not equal to 1 (i.e.: results are not identical).

Num. of sample	ARI	Abs. value of difference in TD	Relative error of TD in %
2	0.901791	0.000323	0.260200
4	0.836160	0.000093	0.074600
7	0.854687	-0.000186	0.150500
10	0.745988	-0.000197	0.157200
11	0.825592	0.000103	0.081600
13	0.841118	0.000369	0.292500
14	0.812377	-0.000358	0.290100
16	0.804698	-0.000393	0.314700
17	0.817172	-0.000106	0.085000
19	0.964368	-0.000029	0.023400
22	0.839646	0.000333	0.265700
26	0.907451	0.000029	0.023000
28	0.846649	0.000154	0.122500
32	0.896000	-0.000039	0.031300
33	0.909806	0.000105	0.083600
34	0.903097	0.000036	0.028500
38	0.781444	0.000432	0.342900
39	0.713274	0.000058	0.046300
42	0.878302	0.000124	0.100400
43	0.803226	-0.000328	0.267200
45	0.828265	0.000089	0.071100
46	0.899587	-0.000058	0.046400
47	0.914852	0.000142	0.114400
51	0.739063	0.000307	0.249000
52	0.872542	-0.000280	0.227200
56	0.882931	-0.000105	0.084100
57	0.930081	0.000424	0.337300
64	0.896909	0.000071	0.057100
65	0.892653	0.000656	0.522400
71	0.856985	-0.000074	0.058500
72	0.747788	0.000517	0.413500
75	0.884916	0.000231	0.184300
78	0.865846	-0.000233	0.186300
81	0.930035	0.000109	0.087700
83	0.840473	0.000043	0.034200
97	0.888031	0.000032	0.025900
98	0.766460	-0.000325	0.261700

Supplementary table 1: Table for accuracy test using $float$ as data type

Num. of sample	ARI	Abs. value of difference in TD	Relative error of TD in %
5	0.944036	-0.000070	0.029600
10	0.857820	0.000140	0.059500
20	0.937719	-0.000170	0.072000
22	0.924597	-0.000091	0.038800
50	0.914693	-0.000075	0.031500
53	0.903764	-0.000112	0.047700
56	0.971737	0.000009	0.003700
61	0.898369	0.000028	0.011600
62	0.766126	0.000532	0.224000

Supplementary table 2: Table for accuracy test using $double$ as data type

Using *f*loat 37 out of the 100 tests exhibited different results. Nevertheless, the maximum relative error in the value of the optimization function is 0.52% and even in this case, the difference in TD values is positive (0.000656) which indicates that *s*cellpam obtained a smaller value of TD (a better solution) than *c*luster. In the cases in which *s*cellpam obtained a larger value of TD than *c*luster, the worst result shows a 0.26% of relative error.

Using *d*ouble as data type results are better. Only 9 out of 100 samples exhibited different results, being the highest difference in favor of *s*cellpam the one which relative error of 0.22%. On the other hand, the highest difference in favor of *c*luster gives a relative error of 0.07%.

This shows that, even if the implementation in package *c*luster is considered as the reference, it not always gives the best results in terms of lowest value of TD because of loss of precision in the implementation.

The final recommendation is that in critical cases the user should get a sample of the data to asses, doing experiments similar to the one proposed here, to what extent the expected error is or not beyond acceptable limits.
